# The independent effects of isolated high-intensity interval training modalities on body composition and adiposity indices in overweight or obese adults: a systematic review and meta-analysis

**DOI:** 10.3389/fphys.2026.1791740

**Published:** 2026-05-29

**Authors:** Jingyou Zhong, Yanhao Wang, Hongliang Wu, Huangkun Chen, Ming Li

**Affiliations:** School of Physical Education and Sport Science, Fujian Normal University, Fuzhou, Fujian, China

**Keywords:** body composition, body fat percentage, high-intensity interval training, obesity, overweight

## Abstract

**Objective:**

To assess the efficacy of different HIIT types (e.g., running vs. cycling) on weight, BMI, and body fat percentage (BF%) in individuals with obesity, while determining how duration and intensity moderate these effects for tailored prescription.

**Methods:**

Following PRISMA protocols, we searched databases such as Embase and Cochrane through August 2025. Eligible Randomized Controlled Trials (RCTs) compared HIIT against controls or moderate-intensity training. Data extraction and bias assessment (Cochrane tool) were conducted in duplicate. We synthesized data using Stata and RevMan software to determine Mean Differences (MD) and heterogeneity (*I*^2^).

**Results:**

Fourteen studies met inclusion criteria. Pooled analysis showed HIIT significantly lowered BMI and BF% but not overall weight. Running-based interventions showed a trend toward greater improvements compared to cycling. Subgroup data suggested that short-term (3–5 weeks), frequent training (> 3x/week) at 80%-89% HRpeak (Zone 4) may favor BMI reduction. In contrast, BF% loss appeared most pronounced with longer protocols (> 12 weeks) at ≥90% HRpeak (Zone 5) performed thrice weekly, especially in men aged 30-60. However, due to small sample sizes and heterogeneity, these potentially optimal parameters should be interpreted with caution.

**Conclusion:**

Running-based HIIT effectively improves body composition. Specific parameter configurations (e.g., Z4 for BMI reduction, Z5 for fat loss) demonstrate preliminary positive trends that support the future development of goal-specific exercise guidelines, though current evidence is insufficient to establish an absolute ‘optimal’ prescription.

**Systematic review registration:**

https://www.crd.york.ac.uk/PROSPERO/, identifier CRD420251163797.

## 1 Introduction

Overweight and obesity constitute a critical global public health challenge, associated with a markedly elevated risk of diverse chronic pathologies, including cardiovascular disease, type 2 diabetes mellitus, and specific malignancies (Poon et al., 2024). As a pivotal hallmark of metabolic syndrome, obesity is inextricably linked to the accumulation of visceral adipose tissue, which subsequently exacerbates insulin resistance and dysregulates lipid metabolism (Jiang et al., 2014). Furthermore, obesity is frequently implicated in the etiology of non-alcoholic fatty liver disease (NAFLD), driven by hepatic lipid deposition and endoplasmic reticulum stress, with the potential to progress toward hepatic fibrosis or cirrhosis (Ernst and Sinal, 2010). Elevated circulating levels of adipokines, such as chemerin, are intimately associated with the pathogenesis of metabolic perturbations in overweight and obese individuals, thereby augmenting inflammatory responses and cardiovascular risk. Among the female demographic, obesity correlates with polycystic ovary syndrome (PCOS), precipitating hyperandrogenism, ovulatory dysfunction, and psychological sequelae such as anxiety and depression. Moreover, the obese state is characterized by chronic low-grade systemic inflammation, manifested by upregulated levels of proinflammatory cytokines (e.g., tumor necrosis factor-α [TNF-α] and interleukin-6 [lL-6]) and a dysregulation of adipokines (e.g., leptin and adiponectin), which may induce aberrant bone turnover and heighten the risk of osteoporosis (Forte et al., 2023). To maintain optimal health, the World Health Organization (WHO) advocates that adults engage in a minimum of 150 minutes of moderate-intensity or 75 minutes of vigorous-intensity physical activity per week (Bull et al., 2020). Notwithstanding these guidelines, a substantial proportion of the global adult population fails to adhere to these recommendations, with primary impediments typically encompassing time constraint slack of motivation, and poor adherence (Silveira et al., 2022). Although high-intensity interval training (HIIT) has been widely promoted as a public health strategy in recent years, its definition in the existing literature is often overly vague and severely lacks physiological rationale. Currently, many studies featuring extremely short intervention periods (e.g., comprising only 1 to 6 training sessions, or lasting a mere 3 to 5 weeks) are inappropriately labeled as HIIT. However, such brief durations are fundamentally insufficient to induce long-term physiological adaptations in the body. As noted in the research by Ahmad Alkhatib et al (Alkhatib, 2023), in the absence of the adaptive benefits derived from long-term training, these short-term interventions can at best be termed “high-intensity exercise,” and do not warrant the full designation of HIIT.

Examined from a rigorous exercise physiology perspective, HIIT is not a novel miracle therapy; essentially, it is a training modality that places the body within the severe exercise intensity domain. As early as the beginning of the 20th century, exercise physiology had already elucidated the specific physiological responses associated with exercising in this intensity domain (such as rapid elevations in blood lactate concentration, alongside significant alterations in oxygen uptake and respiratory quotient) (Hill et al., 1924). Therefore, abandoning the conventional practice of relying on vague definitions based solely on percentages of heart rate or oxygen uptake, the present study strictly defines HIIT as an interval exercise protocol performed within the severe exercise intensity domain, necessitating a sufficiently long intervention period to induce multiple long-term metabolic and cardiovascular adaptations. Clarifying this intrinsic nature is paramount for objectively evaluating the true efficacy of HIIT in overweight or obese populations at risk for multiple chronic comorbidities.

The physiological adaptations induced by HIIT are multidimensional and distinctly time-dependent, characterized by the effective improvement of cardiorespiratory fitness and metabolic health markers despite a reduction in total exercise volume. In the acute phase, HIIT elicits a profound neuroendocrine response characterized by a surge in catecholamines, which rapidly upregulates lipolysis and significantly increases excess post-exercise oxygen consumption (EPOC) (Gibala et al., 2012). Metabolically, even short-term HIIT interventions (2–4 weeks) are sufficient to enhance peripheral insulin sensitivity, augment skeletal muscle glucose uptake, and favorably modulate the hormonal milieu, such as by increasing adiponectin and suppressing pro-inflammatory leptin levels. Cardiovascularly, medium-term adaptations (4–8 weeks) encompass the upregulation of endothelial nitric oxide synthase and the improvement of vascular compliance, which collectively drive enhancements in cardiorespiratory fitness and VO_2_max (Gibala et al., 2012).

Importantly, structural adaptations associated with obesity outcomes necessitate long-term interventions. While the initial phase of training (typically ≤ 4 weeks) often results in only minor fluctuations in overall body mass—likely due to the simultaneous expansion of plasma volume and the preservation of lean body mass—longer-term adaptations (8–12 weeks) are indispensable for achieving clinically significant reductions in BMI and body fat percentage (BF%) (Batacan et al., 2017).

Notwithstanding the promising potential of HIIT in ameliorating body composition, the extant evidence remains limited. First, a preponderance of existing reviews has primarily concentrated on the impact of HIIT on cardio respiratory fitness or risk factors for metabolic syndrome; conversely, there is a paucity of research systematically evaluating the differential effects of distinct HIIT modalities (e.g.cycling, running, and circuit training) on body composition specifically within overweight or obese cohorts (Batacan et al., 2017). Furthermore, while digital health interventions and nudging strategies have been substantiated to effectively enhance physical activity adherence, the synergistic effects of integrating these strategies with HIIT protocols have yet to be comprehensively evaluated (Leppe-Zamora et al., 2025; Zhang et al., 2025).

It is particularly noteworthy that obesity is intimately associated with sedentary behavior and physical inactivity (Haghjoo et al., 2022). Evidence derived from objective device based measurements demonstrates a positive correlation between sedentary time and obesity indices, whereas interrupting sedentary bouts to engage in moderate to-vigorous physical activity (MVPA) is associated with reductions in body fat (Gába et al., 2025). Furthermore, a multitude of studies emphasize that multi-component lifestyle interventions, integrating dietary restriction with physical activity, are efficacious in reducing body weight and adiposity (Rahimi et al., 2022; Heath et al., 2024). Nevertheless, a systematic evaluation and synthesis regarding the specific efficacy of HIIT on body composition in overweight or obese adults-whether deployed as an isolated intervention or as part of a combined regimen-remains lacking.

Furthermore, it must be clarified that this systematic review is designed to strictly isolate and evaluate the independent mechanical and metabolic effects of pure exercise intervention. While the prevention and management of chronic comorbidities—such as obesity—typically necessitate lifestyle interventions that integrate both nutritional and physical activity components, it remains challenging to attribute observed benefits to any single lifestyle factor within such complex, multi-component protocols.

Consequently, the scope of this study was deliberately narrowed to focus exclusively on HIIT as a standalone intervention. Studies combining HIIT with dietary restriction, specific nutritional supplements, or other multifaceted lifestyle modifications were strictly excluded. This rigorous inclusion criteria design serves to precisely disentangle the interference of confounding variables, such as diet and nutrition, thereby allowing for an objective assessment of the true independent impact of HIIT on body composition in overweight and obese populations.

## 2 Methods

### 2.1 Literature search strategy

This systematic review and meta-analysis was conducted in strict accordance with the Preferred Reporting Items for Systematic Reviews and Meta-Analyses (PRISMA) guidelines. The study protocol was prospectively registered with the International Prospective Register of Systematic Reviews (PROSPERO) under registration number CRD420251163797. A comprehensive computerized search was performed across five electronic databases—PubMed, Web of Science, Embase, the Cochrane Library, and EBSCO—covering the period from their respective inceptions to August 2, 2025. The specific search strategy employed a combination of medical subject headings (MeSH) and free-text terms using Boolean operators, as follows: (“High Intensity Interval Training” OR “Interval Training, High-Intensity” OR “Training, High-Intensity Interval” OR “Exercise, High-Intensity Intermittent” OR “Sprint Interval Training”) AND (“overweight” OR “Obesity” OR “obese”) AND (“middle-aged” OR “Adult”) AND (“Body Compositions” OR “Composition, Body” OR “body fat percentage” OR “fat mass” OR “body weight”).

### 2.2 Study selection

Initial deduplication of the retrieved records was performed by one investigator (JY) utilizing EndNote X9 reference management software. Subsequently, two investigators (HK and YH) independently screened the titles and abstracts of the remaining records against predetermined inclusion and exclusion criteria. Articles deemed potentially eligible, or those for which a decision could not be rendered based solely on the title and abstract, were subjected to a full-text assessment. Throughout the screening process, any discrepancies arising between the two primary reviewers were resolved through consultation with a third investigator (JY) to achieve consensus, thereby ensuring the objectivity and consistency of the final selection.

### 2.3 Inclusion and exclusion criteria

Inclusion Criteria Studies were included in the meta-analysis if they satisfied the following criteria: 1) Participants: Adults aged ≥18 years classified as overweight or obese; To isolate the effects of the intervention on the general overweight population, individuals with pathological obesity (secondary to underlying disease), significant comorbidities, or those classified as elite athletes were excluded; 2) Interventions and Comparators: The experimental group performed HIIT. The control group received no exercise intervention, standard care, or engaged in alternative HIIT modalities; 3) Outcomes: The study reported at least one primary outcome measure related to body composition or adiposity, specifically: body weight, BMI or BF%; 4) Study Design: The investigation was strictly limited to Randomized Controlled Trials (RCTs).

Exclusion Criteria Studies were excluded based on the following grounds: 1) Data Unavailability: Relevant outcome data were insufficient for extraction (e.g., absence of baseline or post-intervention Mean ± Standard Deviation [SD]); 2) Publication Type: The record was a conference abstract, review article, meta-analysis, or irrelevant to the specific research topic; 3) Redundancy and Design: Duplicate publications or non-randomized experimental designs were identified.

#### 2.3.1 Operational definitions of HIIT sub types

To address the heterogeneity in intensity reporting across primary studies, this meta-analysis stratified HIIT into specific sub types based on the percentage of peak heart rate (HRpeak), in alignment with the standardized five-zone exercise intensity model:

Zone 4 (Z4) Interval Training: Refers to training protocols where the intensity during the work interval is maintained below 90% of HRpeak (typically falling within the 80%-89% range) (Mann et al., 2013). This zone corresponds to the physiological domains of the lactate threshold and maximal aerobic power. At this intensity, the physiological equilibrium between lactate production and clearance is continuously challenged.

Zone 5 (Z5) Interval Training: Refers to training protocols where the intensity during the work interval equals or exceeds 90% of HRpeak (> 90%) (Mann et al., 2013). This zone represents the anaerobic capacity domain, where energy provision is predominantly reliant on anaerobic glycolysis, precipitating a rapid accumulation of metabolic byproducts and inducing acute physiological stress.

### 2.4 Data extraction

Data extraction was performed independently by two investigators (HK and YH), who had undergone rigorous methodological training, utilizing a standardized data extraction form. Following cross-verification, any discrepancies arising were adjudicated by a third investigator (JY) through discussion and reference to the original texts. The extracted variables encompassed: 1) study characteristics (first author, publication year, and study design); 2) participant demographics (sex, age, and sample size); 3) intervention protocols (HIIT modality [e.g., running, cycling], intensity zone, frequency, and duration); 4) comparator type (no exercise or MICT); and 5) final outcome measures. Studies with irretrievable missing data were excluded from the analysis (Cumpston et al., 2019).

### 2.5 Quality assessment of included studies

The methodological quality of the included studies was systematically assessed using the Cochrane Risk of Bias Tool. The evaluation encompassed six distinct domains (Sterne et al., 2019), comprising: selection bias (evaluated via two core indicators: random sequence generation and allocation concealment), performance bias, detection bias, attrition bias, reporting bias, and other sources of bias. In strict alignment with standard Cochrane recommendations, the overall risk of bias for each study was determined based on qualitative domain-level judgments rather than a numerical scoring system (Zhang et al., 2019).

### 2.6 Statistical analysis

Statistical analyses were conducted utilizing Review Manager 5.4 (The Cochrane Collaboration, Oxford, UK) and Stata 16.0 (StataCorp, College Station, TX, USA). Specifically, Review Manager 5.4 was employed to generate risk of bias visualizations and forest plots, whereas Stata 16.0 was utilized to construct funnel plots (Peters et al., 2008) and execute heterogeneity analyses (Nagashima et al., 2019).

Given that the outcome measures included in this study were continuous variables the Mean Difference (MD) were adopted as the effect size metrics (Andrade, 2020). Results were expressed with 95% Confidence Intervals (CI) to indicate the precision of the estimates. HeterogeneityC among studies was assessed via the Chi-square test (P-value) and the *I*^2^ statistic (Higgins and Thompson, 2002). The *I^2^* value ranges from 0% to 100%. A fixed-effects model was applied to pool effect sizes when heterogeneity was low (*I*^2^ < 50%).Conversely, a random-effects model was adopted when moderate heterogeneity was indicated (*I*^2^ > 50%). In instances where *I*^2^ >75%, signifying substantial heterogeneity, subgroup analyses and meta-regression were conducted to investigate potential sources of heterogeneity (Egger et al., 1997).

## 3 Results

### 3.1 Literature search results

**Figure 1:**
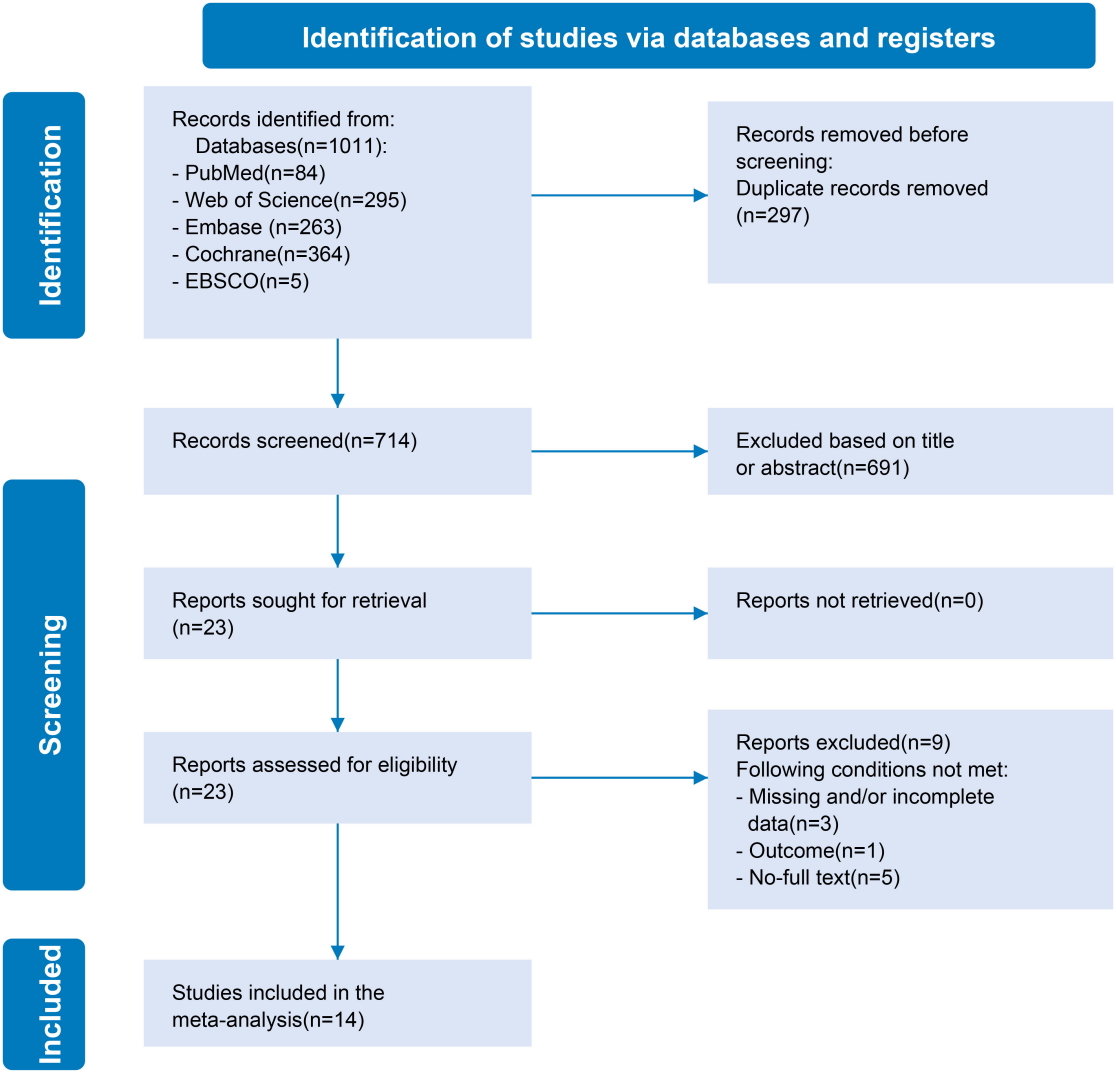
Literature screening flowchart.

A systematic search conducted across five major databases (PubMed, Web of Science, Embase, the Cochrane Library, and EBSCO) yielded an initial identification of 1,011 potentially relevant records. Following the importation of these citations into EndNote reference management software and the subsequent removal of duplicates, 714 unique records remained. During the preliminary screening phase, titles, abstracts, and keywords were scrutinized, resulting in the exclusion of 691 records due to obvious divergence from the research topic, incompatible outcome measures, or ineligible study populations. Consequently, 23 articles advanced to the full-text assessment stage. Finally, following a comprehensive review against the pre-established eligibility criteria, 14 studies (Armannia et al., 2022; Cao et al., 2024; Couvert et al., 2024; D’Alleva et al., 2023; Fisher et al., 2015; Kong et al., 2016; Lu et al., 2021; Martins et al., 2016; Sañudo et al., 2018; Smith-Ryan et al., 2015; Song et al., 2024; Tang et al., 2022; Vaccari et al., 2020; Xu et al., 2022) were identified as satisfying all inclusion requirements and were subsequently incorporated into the final meta-analysis. The detailed flow of the literature selection process is illustrated in **Figure 1**.

### 3.2 Characteristics and quality assessment of included studies

**Figure 2:**
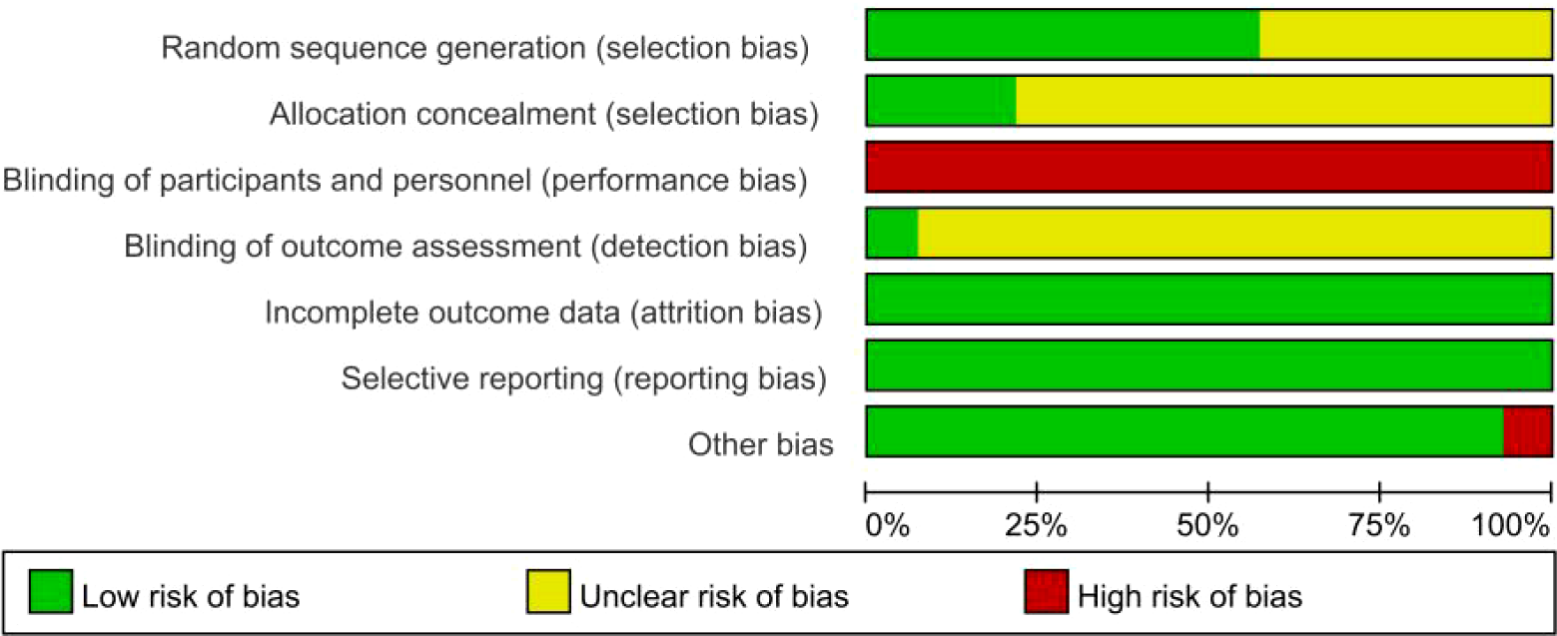
Bias assessment chart of the included.

**Figure 3:**
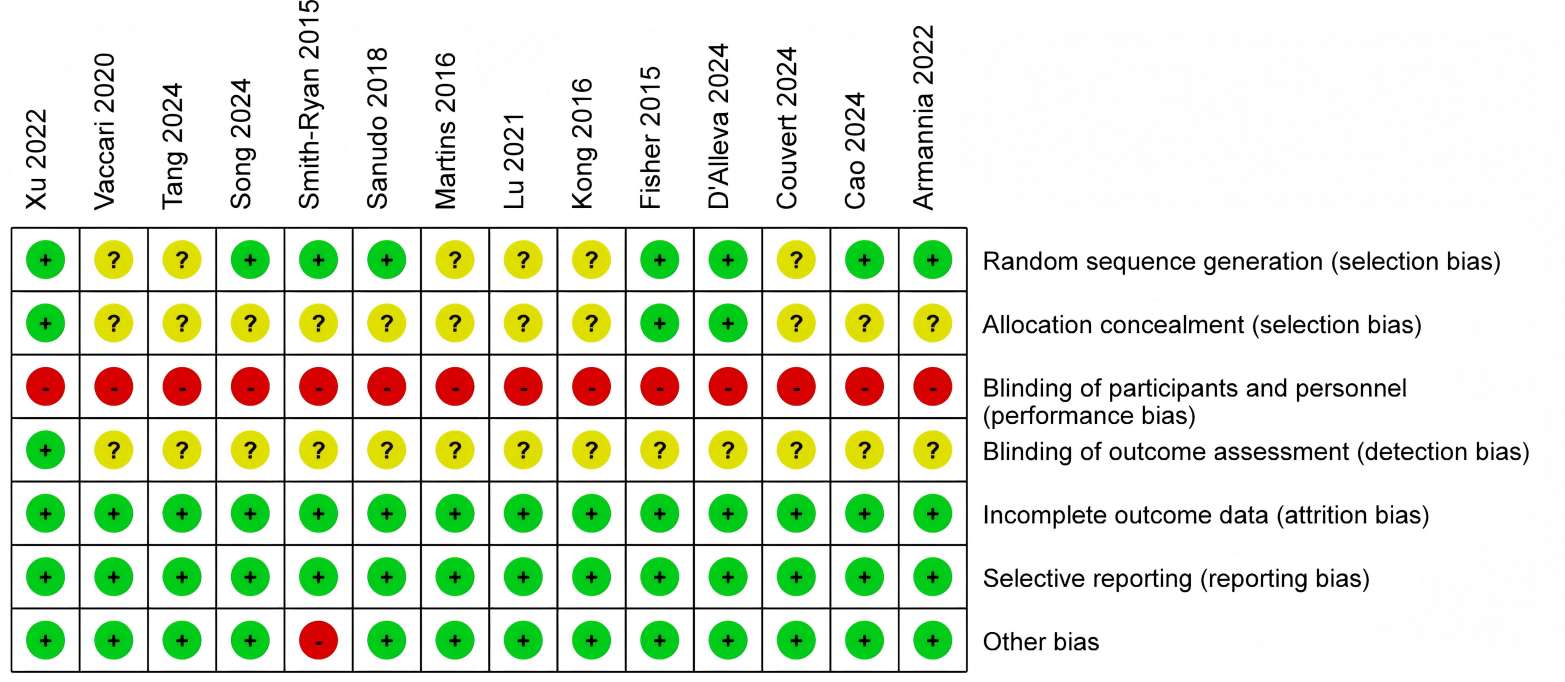
Summary graph of bias in the included.

A total of 14 studies (26—39), all employing a RCTs design, were included in the quantitative synthesis. The cumulative sample size comprised 245 participants. The detailed baseline characteristics of these studies are summarized in **Table 1**. Regarding the methodological quality assessment, three studies were categorized as having a low risk of bias, nine as moderate risk, and two as high risk. Consequently, the overall methodological quality of the included literature was predominantly characterized as “moderate risk of bias,” indicating the presence of potential methodological limitations in a substantial proportion of the included studies. The specific details of the risk of bias assessment are visually presented in **Figures 2**, **3**.

**Table 1:** Baseline characteristics of the included.

Study	Year	Study subjects			Intervention measures					Control group	Outcome measures
		HIIT sample size	M/F	Age (Mean± SD)	Modality	Freq (sessions/wk)	Duration (weeks)	HIIT protocol	Intervention		
Armann	2022	12	6/6	45.33 ± 3.55	Running	3	8	Week 1: 30s (65% max HR), 2 reps. Weeks after: +5% intensity, +30s weekly; +1 rep q2w.	Z4	MICT	A､B､C
Cao 1	2024	13	8/5	23.15 ± 2.27	Functional Training	3	12	each with 4×30s all-out full-body exercises (30s rest within set), 1min rest between sets	NR	Daily activities	A､C
Cao 2	2024	12	7/5	21.54 ± 1.79	Running	3	12	4×30s running each (30s rest within set), 1min rest between sets, intensity increasing per cycle.	NR	Daily activities	A､C
Couvert	2024	8	8/0	52.9 ± 10.3	Cycling	3	12	10×45s high-intensity exercise (80-85% max heart rate) with 90s active recovery	Z4	HIIT	A､B
D’Alleva	2024	16	16/0	38.3 ± 7.1	Running	3	12	5-7×2-min high-intensity (95% peak oxygen uptake) with 1-min low-intensity recovery	Z5	MICT	A､B､C
Fisher	2015	15	15/0	20.0 ± 1.5	Cycling	3	6	4 sets×30s high-intensity (85% peak anaerobic power)	Z4	MICT	A､B､C
Kong	2016	13	0/13	21.5 ± 4.0	Cycling	4	5	60 sets×8s high-intensity cycling with 12s rest intervals	NR	MICT	A､B､C
Lu	2021	10	0/10	20.7 ± 0.6	Running	3	12	30s max shuttle run (20m)/30s rest, 4 sets	Z5	HIIT	A､B､C
Martins	2016	13	4/9	33.9 ± 7.8	Cycling	3	12	8s sprint + 12s recovery, total duration 20min (energy expenditure 250kcal)	Z4	MICT	A
Sañudo	2018	14	4/10	35.3 ± 7.8	Cycling	3	8	6–10 sets×1min HIIT (90% peak heart rate)	Z4	Daily activities	A､C
Smith-Ryan 1	2015	10	10/0	36.5 ± 12.3	Cycling	3	3	0×1min high-intensity cycling (90% peak power output)	Z5	Daily activities	C
Smith-Ryan 2	2015	10	10/0	40.6 ± 12.1	Cycling	3	3	5×2min cycling (intensity 80%-100% peak oxygen uptake)	Z5	Daily activities	C
Song	2024	20	10/10	21.75 ± 1.99	Cycling	3	8	4 sets×4min high-intensity (85-90% max heart rate)	Z4	MICT	A､B､C
Tang	2024	13	3/10	42.2 ± 5.7	Swimming	3	6	12×30s high-intensity swimming (75-90% max heart rate in week 1, 95% max heart rate from week 2)	Z5	MICT	A､B､C
Vaccari	2020	16	9/7	40.1 ± 0.4	Running	3	12	3–7 sets×3min high-intensity walking (100% peak oxygen uptake)	Z5	MICT	A､B
Xu 1	2022	16	7/9	NR	Running	5	4	5 sets×3min high-intensity (80% VO_2_max) and 30% of their daily recommended energy intake (approximately 500–1000 kcal) on 2 non-consecutive days every week	Z4	HIIT	A､B､C
Xu 2	2022	16	6/10	NR	Running	5	4	5 sets×3min high-intensity (80% VO_2_max) and 70% of their estimated energy requirements every day	Z4	HIIT	A､B､C

HIIT, High-Intensity Interval Training; MICT, Moderate-Intensity Continuous Training. A, Body Weight; B, Body Mass Index; C, Body Fat Percentage. The numerals 1 and 2 denote distinct HIIT protocols conducted within the same study; NR, Not Reported.

### 3.3 Meta-analysis

#### 3.3.1 Effect of HIIT on body weight

**Figure 4:**
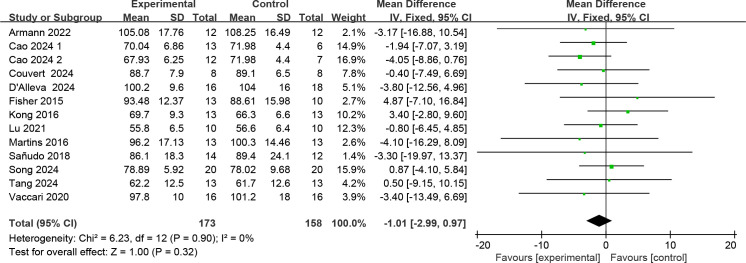
Forest plot illustrating the effects of HIIT on body weight in overweight or obese adult.

A total of 13 studies were synthesized to evaluate the impact of HIIT on body weight. As illustrated in **Figure 4**, the included studies exhibited negligible heterogeneity (*I*^2^ = 0%, *P* =0.90); consequently, a fixed-effects model was employed for the analysis. The aggregated results yielded an overall *MD* of -1.18 (95% *CI*: -2.99 to 0.97, *P*=0.32). These findings indicate that the effect of HIIT on body weight reduction among overweight or obese adults did not reach statistical significance.

As illustrated in **Table 2**, subgroup analyses demonstrated that the intervention effects on body weight did not vary significantly across different stratifications, including exercise modality, intensity zone, intervention duration, sex, frequency, age and control group type (all *P* > 0.05).

**Table 2:** Subgroup analysis based on the results of the meta-analysis.

Subgroup	MD	95% CI	P-value	I² (%)	P-value (heterogeneity)
Exercise Mode					
Cycling	2.27	-0.84, 5.39	0.15	0	0.88
Running	-1.4	-5.13, 2.34	0.46	0	0.98
Swimming	0.5	-9.15, 10.15	NA	NA	NA
Functional Training	-1.94	-6.37, 2.49	NA	NA	NA
Intensity					
Z4	1.15	-2.18, 4.49	0.58	0	0.86
Z5	-0.34	-3.91, 3.22	0.85	0	0.89
NR	-0.33	-3.82, 3.16	0.85	0	0.36
Duration					
Short (3–5 weeks)	3.4	-2.80, 9.60	NA	NA	NA
Medium (6–8 weeks)	0.68	-3.18, 4.54	0.73	0	0.91
Long (12+ weeks)	-0.62	-3.10, 1.85	0.62	0	0.88
Gender					
Male	-0.59	-5.59, 4.42	0.82	0	0.52
Female	1.11	-3.07, 5.28	0.6	0	0.33
Mixed	-2.03	-4.37, 0.31	0.09	0	0.91
Age					
Age < 30 years	-0.9	-3.07, 1.28	0.42	10	0.35
Age 30–60 years	-2.05	-5.85, 1.75	0.29	0	0.99
Training Frequency					
Frequency = 3	-1.65	-3.64, 0.33	0.1	0	0.96
Frequency > 3	3.4	-2.80, 9.60	NA	NA	NA
Control Group Type					
MICT	0.34	-2.56, 3.24	0.82	0	0.82
Daily Activities	-3.14	-0.71, 0.72	0.11	0	0.88

MD, Mean Difference; CI, Confidence Interval; I², I-squared statistic for quantifying heterogeneity; NA, Not Applicable/Not Available; NR, Not Reported; Z4 and Z5, specific intensity zones; MICT, Moderate-Intensity Continuous Training; HIIT, High-Intensity Interval Training.

#### 3.3.2 Effect of HIIT on BMI

**Figure 5:**
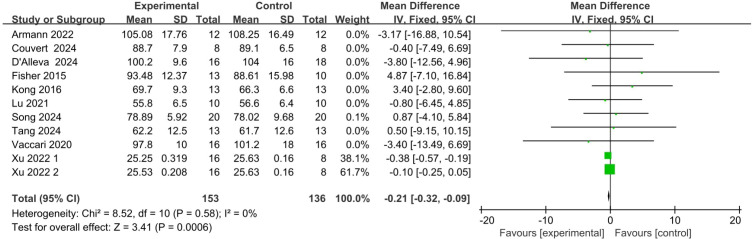
Forest plot illustrating the meta-analysis of the effects of HIIT on BMI in overweight or obese adults.

Eleven studies were synthesized to evaluate the impact of HIIT on BMI. As depicted in **Figure 5**, the included studies exhibited low heterogeneity (*I*^2^ = 0%, *P* =0.58); consequently, a fixed-effects model was adopted for the analysis. The pooled results revealed a statistically significant reduction in BMI with an overall MD of -0.21 (95% *CI*: -0.32 to -0.09, *P*<0.05). These findings indicate that HIIT exerts significant efficacy in reducing BMI among overweight or obese adults.

Subgroup analysis results indicated that running-based HIIT was associated with a significantly greater reduction in BMI among overweight or obese adults compared to cycling (P < 0.05). Protocols utilizing Zone 4 (Z4) intensity appeared significantly more effective in reducing BMI than those in Zone 5 (Z5) (P < 0.05). In terms of duration, short-term interventions (3–5 weeks) yielded a significantly greater reduction in BMI compared to medium- and long-term cycles (P < 0.05). Additionally, a training frequency of > 3 sessions per week was associated with a significantly greater BMI reduction than exactly 3 sessions per week (P < 0.05). Other subgroup analyses revealed that demographic variables such as sex and age had no statistically significant moderating effect on the efficacy of HIIT in lowering BMI (P > 0.05).

To account for clinical heterogeneity among control groups, we performed a subgroup analysis based on the type of comparator (i.e., HIIT vs. MICT/daily activities, and HIIT vs. alternative HIIT modalities). This analysis revealed no significant differences between subgroups (P=0.50, I² = 0%), indicating that the therapeutic efficacy of the primary HIIT intervention remained consistent regardless of the control conditions. Specifically, the mean difference (MD) for HIIT compared to MICT was -0.44 (95% CI: -1.14 to 0.25). When compared to alternative HIIT modalities, the MD was -0.20 (95% CI: -0.32 to -0.08). The overall pooled effect remained statistically significant (MD = -0.21, 95% CI: -0.33 to -0.09, P < 0.01), as presented in **Table 3**.

**Table 3:** Subgroup analysis of the meta-analysis results for BMI.

Subgroup	MD	95% CI	P-value	I² (%)	P-value (heterogeneity)
Exercise Mode					
Cycling	-0.12	-0.86, 0.61	0.74	59	0.06
Running	-0.2	-0.30, -0.10	<0.01	33	0.19
Swimming	-0.5	-3.78, 2.78	0.76	NA	NA
Intensity Zone					
Z4	-0.2	-0.30, -0.10	<0.01	58	0.03
Z5	-0.73	-2.12, 0.66	0.3	0	0.93
Duration					
Short (3–5 weeks)	-0.19	-0.30, -0.09	<0.01	76	0.02
Medium (6–8 weeks)	-0.65	-1.47, 0.17	0.12	0	0.64
Long (12+ weeks)	-0.01	-1.30, 0.17	0.98	18	0.3
Gender					
Male	0.59	-0.91, 2.09	0.44	41	0.18
Female	0.53	-0.86, 1.92	0.45	0	0.38
Mixed	-0.21	-0.31, -0.10	<0.01	41	0.13
Age					
Age < 30 years	-0.33	-1.06, 0.40	0.38	33	0.21
Age 30–60 years	-0.07	-1.40, 1.26	0.92	0	0.43
Training Frequency					
Frequency = 3	-0.47	-1.16, 0.22	0.18	0	0.54
Frequency > 3	-0.19	-0.30, -0.09	<0.01	76	0.02
Control Group Type					
MICT	-0.44	-1.44,0.25	0.21	0	0.52
HIIT	-2.49	-3.12,-1.86	<0.01	88	<0.01

**Figure 6:**
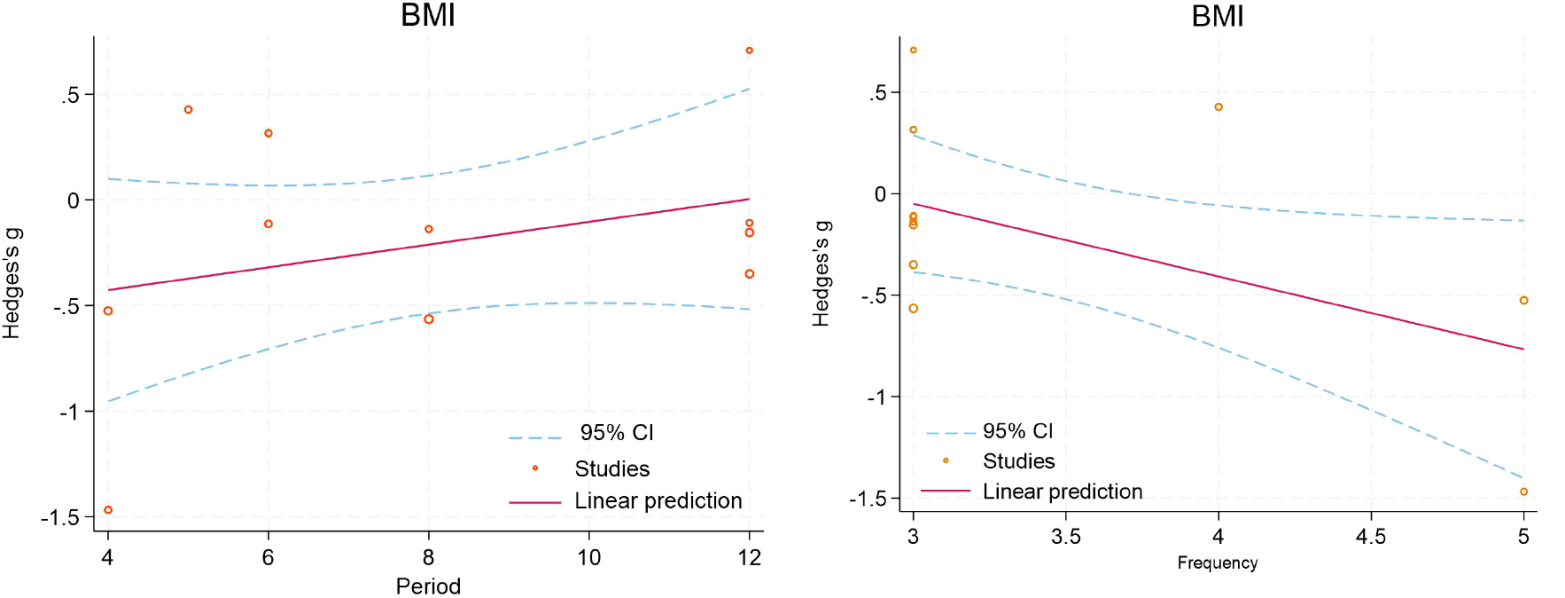
Bubble plot illustrating the meta-regression analysis of BMI.

Regression analysis demonstrated that neither training frequency *(β*=-0.36, 95% *CI*: -0.73 to 0.01) nor intervention duration(*β* =0.05, 95% *CI*: -0.05 to 0.16)served as significant predictors for the efficacy of HIIT in ameliorating BMI among overweight or obese adults (*P* > 0.05), as illustrated in **Figure 6**.

#### 3.3.3 Effect of HIIT on BF%

**Figure 7:**
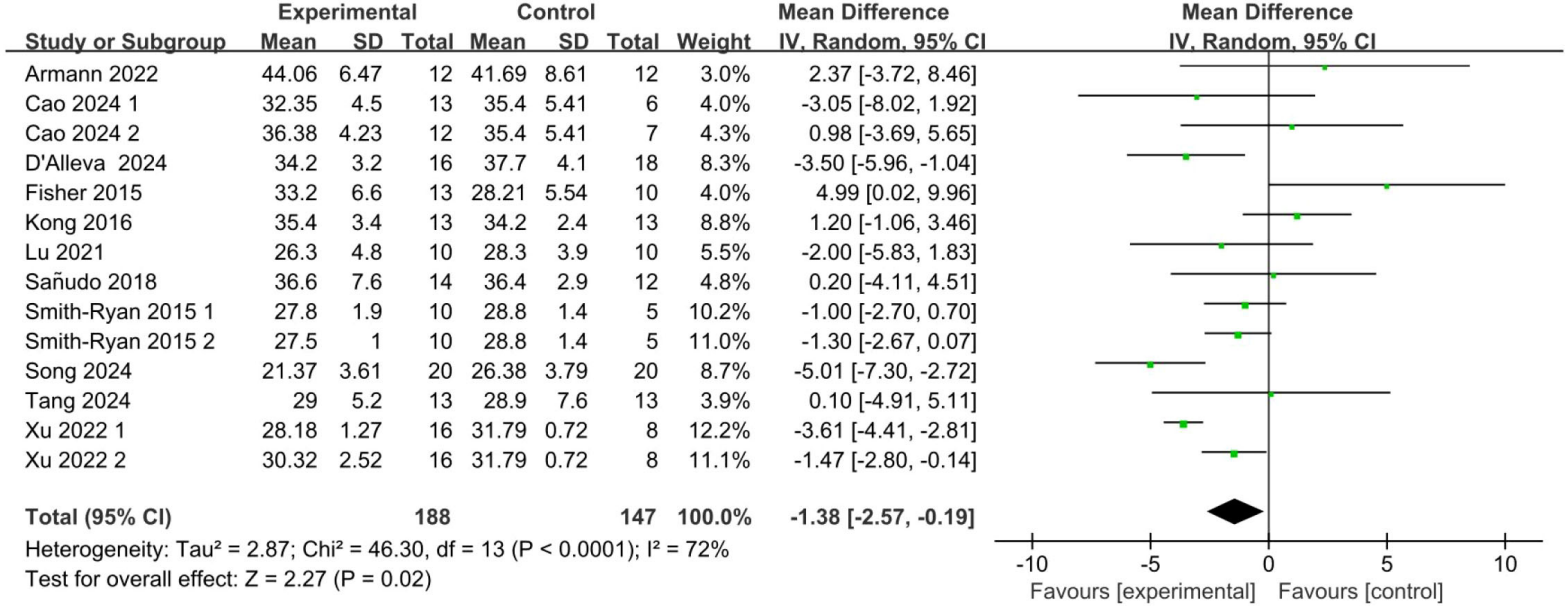
Forest plot illustrating the meta-analysis of the effects of HIIT on BF% in overweight or obese adults.

Fourteen studies were synthesized to investigate the efficacy of HIIT in reducing BF% among overweight or obese adults. As depicted in **Figure 7**, the included studies exhibited moderate-to-substantial heterogeneity (*I*^2^=72%, *P*<0.01); accordingly, a random-effects model was employed for the data synthesis. The pooled analysis revealed an overall MD of -1.38 (95% *CI*: -2.57 to -0.14, *P*<0.05). These findings indicate that HIIT exerts a statistically significant beneficial effect on reducing BF% in this population.

To further explore the potential sources of substantial heterogeneity, subgroup and meta-regression analyses were conducted for the BF% outcome. Subgroup analysis indicated that running-based HIIT showed a significantly more favorable trend in reducing BF% among overweight or obese adults compared to cycling (P < 0.05). Protocols utilizing Z5 intensity appeared significantly more effective in reducing BF% than those in Z4 (P < 0.05). Regarding duration, long-term interventions (12+ weeks) were associated with a significantly greater BF% reduction than short- and medium-term cycles (P < 0.05). Demographically, the efficacy of HIIT in reducing BF% was significantly more pronounced in males than in females (P < 0.05), and in middle-aged adults (30–60 years) compared to younger adults (18–30 years) (P < 0.05). Additionally, a training frequency of exactly 3 sessions per week was associated with a significantly greater BF% reduction than frequencies exceeding 3 sessions per week (P < 0.05). Finally, the reduction in BF% elicited by HIIT was more significant when compared against a daily activities control group than against an MICT control group (P < 0.05), as shown in **Table 4**.

**Table 4:** Subgroup analyses of the meta-analysis results for BF%.

Subgroup	MD	95% CI	P-value	I² (%)	P-value (heterogeneity)
Exercise Mode					
Cycling	-0.68	-2.67, 1.31	0.5	77	0.0007
Running	-2.04	-3.59, -0.50	0.01	68	0.007
Swimming	0.1	-4.91, 5.11	NA	NA	NA
Intensity Zone					
Z4	-1.57	-3.59, 0.45	0.13	81	<0.01
Z5	-1.52	-2.45, -0.59	0.001	0	0.49
NR	-0.01	-2.53, 2.51	0.99	46	0.16
Duration					
Short (3–5 weeks)	-1.41	-2.98, 0.16	0.08	86	<0.01
Medium (6–8 weeks)	0.17	-3.86, 4.20	0.93	78	0.0001
Long (12+ weeks)	-2.17	-4.11, -0.24	0.03	24	0.27
Gender					
Male	-1.3	-2.27, -0.34	0.008	68	0.02
Female	0.37	-1.57, 2.32	0.71	50	0.16
Mixed	-2.98	-3.55, -2.40	<0.01	68	0.003
Age					
Age < 30 years	-0.72	-3.59, 2.15	0.62	78	0.0003
Age 30–60 years	-1.32	-2.32, -0.32	0.01	7	0.37
Training Frequency					
Frequency = 3	-1.75	-2.98, -0.52	0.005	45	0.06
Frequency > 3	-1.49	-3.93, 0.94	0.23	91	<0.01
Control Group Type					
MICT	-0.46	-3.52, 2.59	0.77	80.17	0
Daily Activities	-1.12	-2.08, -0.17	0.02	0	0.74

**Figure 8:**
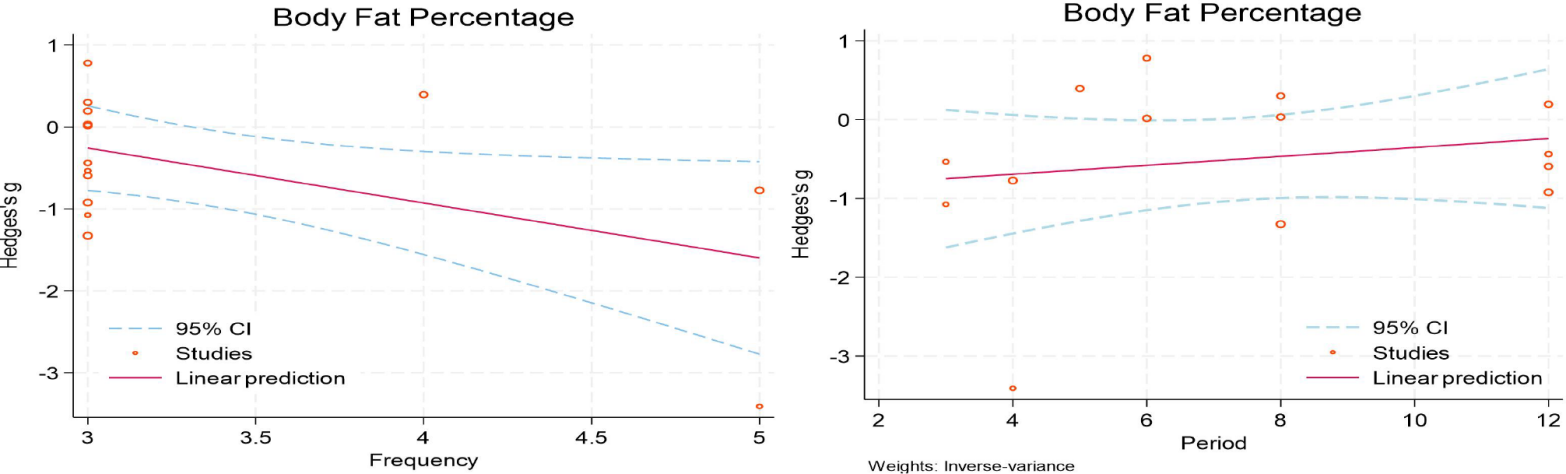
Bubble plot illustrating the meta-regression analysis of BF%.

Meta-regression analysis revealed that neither training frequency(*β* =-0.56, 95% *CI*: -1.19 to -0.08, *P*=0.08) nor intervention duration (*β* =0.06, 95% *CI*: -0.10 to 0.21, *P*=0.47)significantly modulated the efficacy of HIIT in improving BF% among overweight or obese adults (P > 0.05), as illustrated in **Figure 8**.

### 3.4 Sensitivity analysis and risk of publication bias

**Figure 9:**
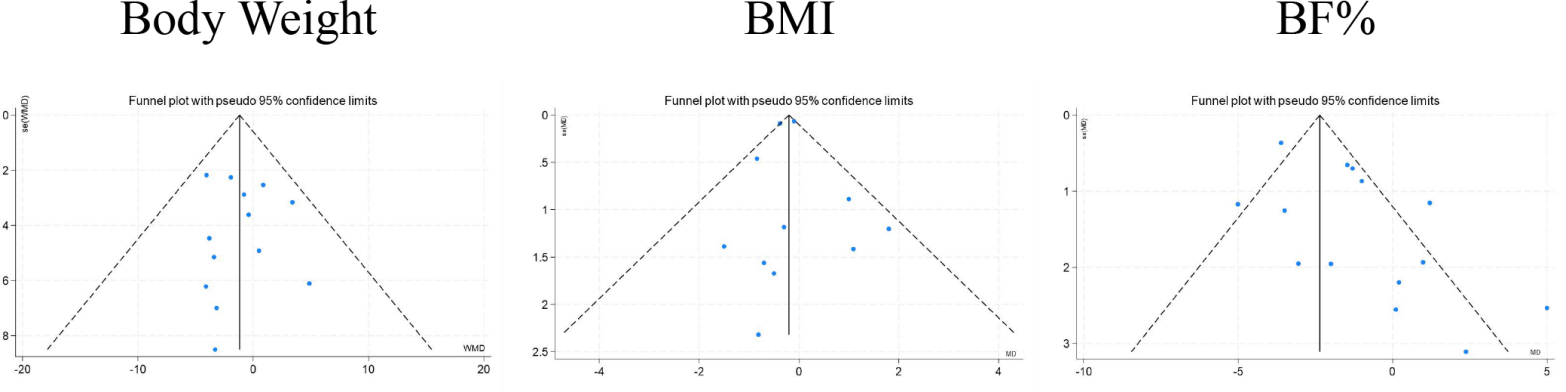
Funnel plot for the assessment of publication bias.

Sensitivity analyses were performed using the leave-one-out method to assess the robustness of the pooled estimates. The results indicated that the pooled effects for body weight and BF% remained largely unchanged after omitting any single study, confirming the stability of these findings. However, the sensitivity analysis for BMI revealed that upon the exclusion of the “Xu 2022” study (Xu et al., 2022), the pooled effect size was substantially altered and the difference was no longer statistically significant (*P* > 0.05). This demonstrates that the current pooled finding regarding HIIT significantly reducing BMI lacks robustness and is highly driven by this single study. Furthermore, visual inspection of the funnel plots revealed that while the majority of the included studies were clustered within the funnel boundaries, the distribution displayed an apparent asymmetry. This lack of symmetry suggests the presence of a certain degree of potential publication bias in the current body of evidence (**Figure 9**).

## 4 Discussion

Based on a meta-analysis synthesizing data from 14 RCTs, the present study systematically evaluated the intervention efficacy of distinct HIIT modalities on body weight, BMI), and BF% in overweight or obese adults. Furthermore, this investigation elucidated the moderating roles of critical training parameters, including training frequency, intensity zones, and intervention duration. The findings demonstrate that while HIIT elicits significant amelioration of BMI and BF% in this population, no statistically significant effect was observed regarding body weight reduction. Collectively, these results provide a robust evidence base for the formulation of precision exercise prescriptions tailored to overweight and obese individuals.

### 4.1 The effect of HIIT on body weight

In the present meta-analysis incorporating 13 RCTs, the results demonstrated that HIIT did not exert a statistically significant impact on body weight reduction among overweight or obese adults. The observed low level of heterogeneity across the included studies indicates a high degree of consistency within the findings. This conclusion aligns with those drawn from a multitude of prior investigations. For instance, a systematic review by Kramer et al. (2023) noted that HIIT did not demonstrate superior efficacy in weight reduction when compared to MICT. Similarly, an 8-week intervention study conducted by Shenoy Basti et al. (2024) indicated that although a decreasing trend in body weight was observed among HIIT participants, neither intragroup nor intergroup comparisons reached statistical significance.

Analyzed through the lens of energy balance, fluctuations in body weight are governed by the dynamic interplay between energy intake and expenditure. The limited efficacy of HIIT in reducing body weight observed in this study may be attributable to multifactorial etiologies. First, the intervention durations in a subset of studies were relatively brief (ranging from 3 to 8 weeks), which may be insufficient to induce a substantial and sustained state of negative energy balance (Jiang et al., 2024). Second, HIIT may precipitate compensatory energy mechanisms, wherein participants inadvertently increase dietary intake to partially offset the exercise-induced energy expenditure (Racil et al., 2023). Furthermore, in the majority of the included studies, dietary intake was not rigorously controlled; consequently, dietary factors, acting as potential confounding variables, may have obscured the true efficacy of HIIT on body weight regulation (Gaweł et al., 2024). Current evidence suggests that low-volume HIIT yields a magnitude of weight loss comparable to that of MICT, with neither modality typically producing clinically substantial reductions in gross body weight when utilized as an isolated intervention (Wewege et al., 2017). However, HIIT demonstrates significant clinical utility in optimizing body composition; specifically, it facilitates a meaningful reduction in BF% and the preservation or accretion of lean body mass, thereby serving as a time-efficient strategy for metabolic health (Sultana et al., 2019).

Subgroup analyses revealed that none of the investigated variables—including exercise modality, intensity configuration, intervention duration, participant sex, age, and training frequency—exerted a statistically significant moderating effect on body weight outcomes (*P* > 0.05). This observation implies that the impact of HIIT on body weight is likely predominantly contingent upon the overall state of energy balance, rather than being governed solely by isolated training parameters. Consequently, future investigations employing more rigorous experimental designs—specifically through the implementation of prolonged intervention durations, stringent dietary controls, and the evaluation of inter-individual variability—are warranted to further elucidate the underlying mechanisms and practical efficacy of HIIT in the domain of weight management.

### 4.2 The effect of HIIT on BMI

In the present meta-analysis incorporating 11 RCTs, the aggregated results demonstrated that HIIT elicited a statistically significant reduction in BMI among overweight or obese adults, characterized by a low degree of inter-study heterogeneity. This finding corroborates conclusions drawn from a multitude of prior investigations. For instance, a 12-week low-volume HIIT study conducted by Reljic et al. (2023) involving patients with metabolic syndrome, as well as a school-based intervention targeting obese children implemented by Cao et al. (2022), both reported favorable improvements in BMI attributable to HIIT protocols.

A mechanistic analysis of the observed BMI improvements suggests that these alterations are predominantly attributable to reductions in adipose tissue mass, rather than significant fluctuations in lean body mass. This inference is substantiated by empirical evidence from primary research. For instance, in a 12-week HIIT intervention conducted by D’Alleva et al (D'Alleva et al., 2023), obese male participants exhibited a statistically significant reduction in BMI that was concomitant with a marked decrease in BF%, whereas lean body mass—an indicator of muscle mass—remained statistically unchanged. These findings compellingly indicate that HIIT achieves effective BMI management primarily by preferentially targeting the reduction of adipose tissue volume and optimizing body composition, rather than inducing weight loss at the expense of muscle catabolism.

To delineate the optimal implementation strategies for HIIT-induced BMI improvement, this study conducted further subgroup analyses, identifying a multidimensional configuration of effective intervention parameters. Regarding exercise modalities, running-based HIIT protocols elicited a significantly superior regulatory effect on BMI compared to cycling-based modalities (*P*<0.05). This disparity is likely attributable to the inherent biomechanical characteristics of running. As a quintessential whole-body weight-bearing exercise, running necessitates the simultaneous activation of the quadriceps, hamstrings, and core musculature, while also recruiting the upper extremities for synergistic participation (Moore, 2016). Consequently, the energy expenditure efficiency per unit of time is significantly elevated compared to cycling (Millet et al., 2009). In terms of exercise intensity, HIIT interventions performed within Z4 demonstrated superior efficacy compared to the higher-intensity Z5 protocols (*P*<0.05) (Hindsø et al., 2022). It is postulated that this phenomenon is associated with the enhanced tolerability of Z4 intensity; participants are more likely to sustain engagement throughout the full intervention period, thereby augmenting overall training adherence (Reljic et al., 2023). With respect to intervention duration and frequency, short-term interventions (3–5 weeks) and high-frequency protocols (> 3 sessions per week) yielded more pronounced improvements in BMI (*P*<0.05) (Ahmad et al., 2023). These findings suggest that short-term, high-frequency HIIT may facilitate rapid weight regulation by upregulating signaling pathways associated with lipolysis, thereby inducing an “acute metabolic adaptation effect” (González-Gálvez et al., 2024; Monsalves-Álvarez et al., 2023). Furthermore, subgroup analyses for sex and age revealed no significant interaction effects (*P* > 0.05) (Youssef et al., 2022), indicating that the beneficial efficacy of HIIT on BMI possesses robust generalizability across diverse demographic cohorts (Racil et al., 2024).

The meta-regression analysis indicated that neither training frequency (*β*=-0.36, *P* > 0.05) nor intervention duration (*β*=0.05, *P* > 0.05) served as significant predictors for the efficacy of HIIT in ameliorating BMI. The minor discrepancy observed between these findings and the conclusions drawn from the subgroup analyses may be attributable to interference from multiple confounding factors. First, the relatively limited sample size of the included studies may have resulted in insufficient statistical power within the regression model to detect significant associations. Second, the majority of primary studies failed to implement rigorous controls over critical variables such as dietary structure and energy intake (Chin et al., 2022). Given the significant interaction effects between dietary factors and exercise interventions, this lack of control may have obscured the true regulatory effects of training frequency and duration (Guo et al., 2022). Consequently, future investigations utilizing larger sample sizes, employing multi-center designs, and enforcing strict control over confounding variables are warranted to further verify the regulatory value of these parameters.

### 4.3 The effect of HIIT on BF%

Based on a meta-analysis incorporating 14 RCTs, the results indicate that HIIT is capable of significantly reducing BF% in overweight or obese adults. While a moderate degree of heterogeneity was observed across the included studies, this conclusion corroborates the findings of a previous meta-analysis conducted by Liu et al. (2025). From a mechanistic perspective, the biological pathways through which HIIT regulates adiposity have been substantiated by a multitude of investigations. First, this training modality significantly enhances the rate of fat oxidation during exercise, thereby facilitating a greater consumption of lipid substrates per unit of time (Sanca-Valeriano et al., 2023). Second, HIIT effectively induces the Excess EPOC effect, sustaining elevated energy metabolism for a prolonged duration following the cessation of exercise, which indirectly promotes lipolysis (Mendelson et al., 2022). Third, long-term HIIT interventions have been shown to ameliorate insulin sensitivity, thereby mitigating lipid accumulation within adipocytes and ultimately leading to a reduction in total body fat content (Cao et al., 2022).

To delineate the determinants influencing the intervention efficacy of HIIT, this study conducted further subgroup analyses, identifying several critical moderating variables. Regarding training modality, running-based HIIT protocols elicited significantly superior reductions in BF% compared to cycling-based modalities (P < 0.05). This disparity may stem from the more comprehensive synergistic contraction of trunk core musculature during running, which not only augments energy expenditure but also facilitates the lipolysis and oxidation of visceral adipose tissue (Li et al., 2022). In terms of exercise intensity, HIIT interventions performed within Z5 demonstrated superior efficacy relative to Z4 protocols (*P*<0.05). It is postulated that higher-intensity stimuli more fully recruit fast-twitch muscle fibers and prolong the duration of post-exercise lipid oxidation, thereby potentiating the adiposity-reducing effects (Trapp et al., 2008). With respect to intervention duration, long-term HIIT (≥ 12 weeks) yielded more pronounced regulatory effects on body fat (*P*<0.05). This suggests that sustained exercise intervention progressively optimizes the activity of lipid-metabolism-related enzymes and mitochondrial function, establishing a robust physiological environment for continuous fat utilization (Yuan et al., 2024; Santana-Oliveira et al., 2025). Regarding participant characteristics, males and individuals aged 30–60 years derived significantly greater benefits from HIIT (*P*<0.05). These observations are likely closely associated with physiological attributes such as the relatively higher muscle mass and basal metabolic rate in males, as well as the relative stability of androgen levels within this specific age cohort (Handelsman et al., 2018). Finally, concerning training frequency, protocols consisting of exactly three sessions per week outperformed those exceeding three sessions (*P*<0.05). This finding implies that a moderate training frequency facilitates an optimal equilibrium between exercise stimulus and physiological recovery, thereby averting the accumulation of fatigue and maladaptive metabolic responses associated with overtraining (Leung et al., 2024; Moreno-Cabañas et al., 2025).

Further meta-regression analysis demonstrated that training frequency exerts a statistically significant influence on the amelioration of BF% (*β*=-0.67, *P* =0.045). This finding aligns with the observations of Liu et al. (2025), who identified a frequency of three sessions per week as optimal for BF% reduction, suggesting that frequencies deviating from this optimum—whether excessive or insufficient—may attenuate the intervention efficacy. In contrast, the impact of intervention duration on BF% did not reach statistical significance (*β*=0.06, *P*=0.47). This lack of significance may be attributable to inconsistent dietary adherence among participants during prolonged study periods (Sheikholeslami-Vatani and Rostamzadeh, 2022). Moreover, a multitude of studies have emphasized that training frequency constitutes a critical determinant in the improvement of body composition (Domaradzki et al., 2022; Wang et al., 2022), thereby further underscoring the imperative of establishing rational training frequencies for effective adiposity regulation.

### 4.4 Limitations

The findings of the present meta-analysis should be interpreted within the context of several inherent limitations. First, substantial methodological heterogeneity characterized the included HIIT protocols, with a distinct lack of standardization regarding exercise modalities, intensity configurations, and recovery intervals. Notwithstanding the subgroup analyses employed to delineate these sources of variance, this heterogeneity may potentially compromise the stability of the pooled outcomes. Second, a subset of primary studies failed to implement rigorous monitoring or reporting of dietary intake; consequently, the confounding influence of dietary factors on the observed alterations in body composition cannot be definitively precluded. Third, the preponderance of included investigations entailed relatively short-term follow-up periods, thereby impeding the evaluation of the long-term sustainability of the therapeutic benefits elicited by HIIT.

The present study has several limitations: 1) Purity of the Intervention vs. Real-World Application During the design phase, to accurately assess the independent effect of HIIT as a sole exercise prescription on body composition, we strictly limited the purity of the intervention. Specifically, we only included RCTs comparing HIIT against a no-exercise control, standard care, or MICT. We acknowledge that while this methodological decision maximized the control of confounding factors and isolated the physiological effects of HIIT itself, it inevitably led to the exclusion of high-quality studies that met other inclusion criteria. Specifically, this meta-analysis failed to include studies that combined HIIT with nutritional supplementation, dietary restructuring, or multi-component lifestyle interventions. Consequently, our findings reflect the net independent effect of HIIT, rather than its synergistic comprehensive effect when combined with other interventions in real-world settings. For instance, evidence suggests that caffeine supplementation can significantly optimize the fat-loss effects of HIIT in obese women, reduce androgen levels, and ameliorate potential side effects such as metabolic endotoxemia and hyperinsulinemia (Alkhatib et al., 2020). Furthermore, from a public health perspective, preventive strategies for multiple long-term health risks (e.g., diabetes and cardiovascular disease) often rely on optimized lifestyle behaviors combining exercise and nutrition, rather than a single exercise modality (Alkhatib, 2023). By overlooking these combined interventions and the broader lifestyle context, our findings may merely represent a conservative estimate of the clinical efficacy achievable through a comprehensive HIIT paradigm. 2) Inclusion of Extremely Short-Term Trials To maximize the sample size of the meta-analysis, we did not strictly exclude studies based on a minimum HIIT intervention duration during the initial screening. This led to the inclusion of trials with extremely short intervention periods (lasting only 3, 4, or 5 weeks). We must critically point out this major flaw. As previously discussed when redefining HIIT, an intervention period of 3 to 5 weeks is fundamentally too short to induce long-term and stable physiological adaptations. For high-risk populations with obesity or multiple chronic metabolic diseases, short-term high-intensity interventions not only struggle to yield substantial health benefits but may also trigger transient metabolic side effects during the early to mid-stages of the intervention (e.g., within the first 8 weeks), including exacerbating metabolic endotoxemia and inducing abnormal spikes in glucose and insulin markers. Physiological evidence indicates that these acute adverse metabolic reactions induced by high-intensity interventions are only effectively mitigated when patients maintain long-term compliance and the body ultimately establishes long-term physiological adaptations. Therefore, the phenomenon observed in the subgroup analysis of this study—where extremely short cycles of 3 to 5 weeks appeared to significantly reduce BMI—must absolutely not be misinterpreted as true adipose tissue depletion or beneficial body composition remodeling. It is highly likely that this is merely fluid loss or temporary acute metabolic fluctuations under severe physiological stress. 3) Ambiguity in Intensity Classification and Protocol Heterogeneity Although we categorized HIIT into Z4 and Z5 based on the percentage of %HRpeak, this classification criterion inherently harbors flaws in retrospective studies. The original studies varied vastly in their reporting of intensity, relying on metrics such as %HRmax, %VO2max, or percentage of peak power output. Inevitably, biases emerged when we uniformly mapped these onto specific heart rate zones. More importantly, the HIIT protocols in some included studies (such as sprint interval training) differed significantly from the traditional definition of HIIT regarding exercise duration and work-to-rest ratios. Merging and analyzing these highly heterogeneous protocols uniformly as HIIT is likely a primary reason for the moderate heterogeneity observed in the BF% analysis. This definitional ambiguity weakens our ability to provide a definitive answer regarding which specific mode or intensity of HIIT is most effective. 4) Weak Robustness of Pooled Results for Certain Outcomes The robustness of the pooled results for some outcome indicators is relatively weak. As revealed by the sensitivity analysis, the conclusion of this study that HIIT can significantly reduce BMI in overweight/obese populations is highly dependent on a single study (Xu 2022) (Xu et al., 2022). Upon excluding this study, the improvement in BMI loses its statistical significance (P > 0.05). This indicates that the current chain of evidence proving HIIT can independently and significantly reduce BMI remains fragile. The clinical heterogeneity present in the existing included literature—regarding intervention intensity, cycle length, sample size, and participant baselines—likely allowed a single study to dominate the overall pooled effect. Thus, the actual therapeutic efficacy of HIIT in reducing BMI must be interpreted with extreme caution.

## 5 Conclusion

**Figure 10:**
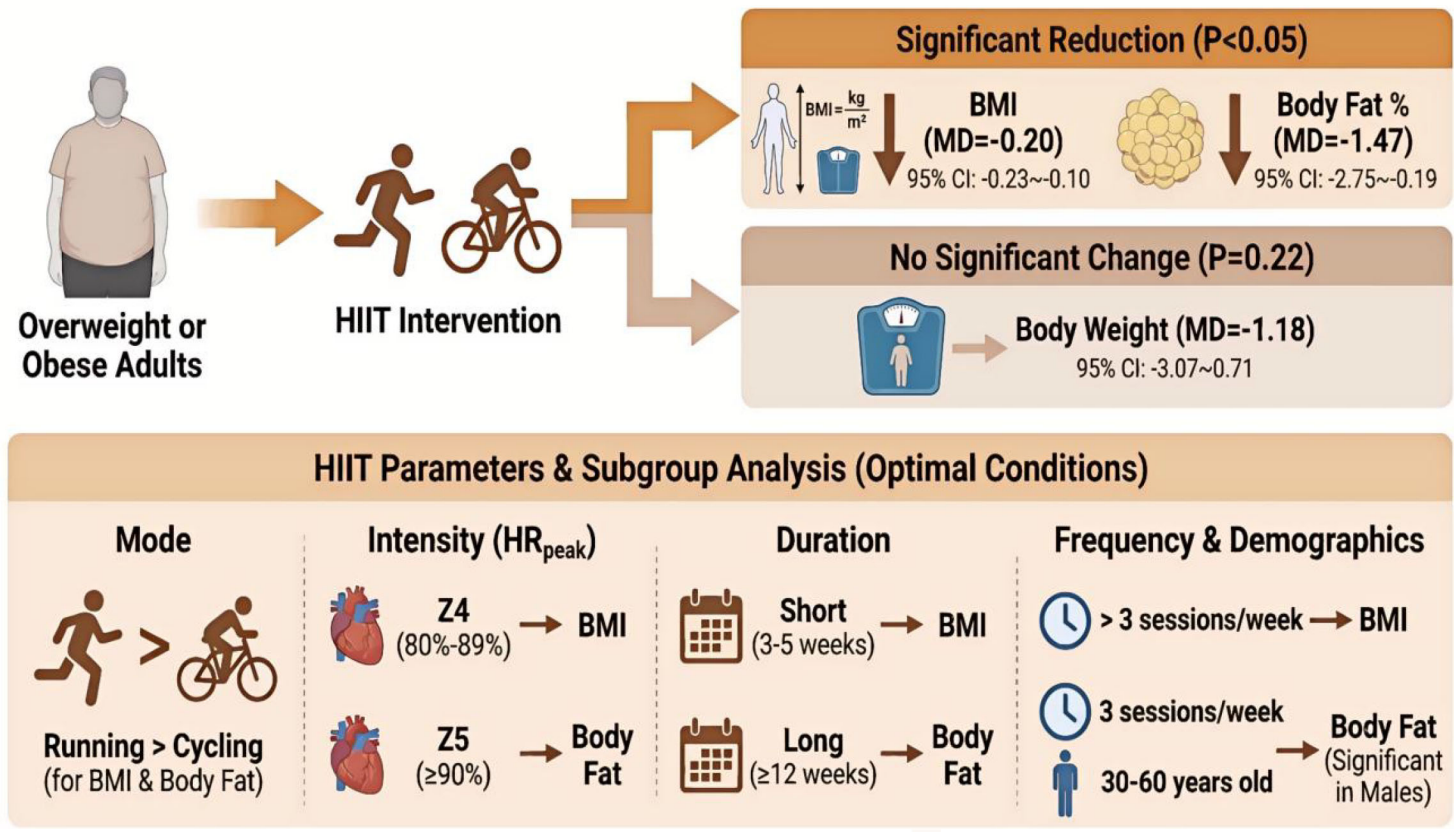
Evidence-based practice recommendations for HIIT to improve body composition in overweight and obese adults.

While this systematic review and meta-analysis confirms that HIIT—particularly running-based modalities—is an effective intervention for improving body composition in overweight or obese adults, with preliminary subgroup data suggesting potential strategic trends for BMI (Zone 4) and BF% (Zone 5) reduction, the limitations of small sample sizes and heterogeneity necessitate a cautious interpretation. Consequently, in clinical translation, these findings should serve as a promising framework to be integrated with the ACSM’s FITT-VP principles for formulating individualized exercise prescriptions (Kanaley et al., 2022) (**Figure 10**), pending further empirical validation from future large-scale, rigorously controlled randomized trials.

## Data Availability

The original contributions presented in the study are included in the article/**Supplementary Material**. Further inquiries can be directed to the corresponding author.
